# A prospective study of frequency of eating restaurant prepared meals and subsequent 9-year risk of all-cause and cardiometabolic mortality in US adults

**DOI:** 10.1371/journal.pone.0191584

**Published:** 2018-01-23

**Authors:** Ashima K. Kant, Barry I. Graubard

**Affiliations:** 1 Dept. of Family, Nutrition, and Exercise Sciences, Queens College of the City University of New York, Flushing, New York, United States of America; 2 Division of Cancer Epidemiology and Genetics, Biostatistics Branch, National Cancer Institute, National Institutes of Health, Bethesda, Maryland, United States of America; Dartmouth College Geisel School of Medicine, UNITED STATES

## Abstract

Restaurant prepared foods are known to be energy-dense and high in fat and sodium, but lower in protective nutrients. There is evidence of higher risk of adiposity, type II diabetes, and heart disease in frequent consumers of restaurant meals. However, the risk of mortality as a long-term health consequence of frequent consumption of restaurant meals has not been examined. We examined the prospective risk of all-cause and coronary heart disease, cerebrovascular disease and diabetes (cardiometabolic) mortality in relation to frequency of eating restaurant prepared meals in a national cohort. We used frequency of eating restaurant prepared meals information collected in the National Health and Nutrition Examination Surveys, conducted from 1999–2004, with mortality follow-up completed through Dec. 31, 2011 (baseline age ≥ 40y; n = 9107). We estimated the relative hazard of all-cause and cardiometabolic mortality associated with weekly frequency of eating restaurant meals using Cox-proportional hazards regression methods to adjust for multiple covariates. All analyses accounted for complex survey design and included sample weights. Over 33% of all respondents reported eating ≥3 restaurant prepared meals/week. In this cohort, 2200 deaths due to all causes and 665 cardiometabolic deaths occurred over a median follow-up of 9 years. The covariate-adjusted hazard ratio of all cause or cardiometabolic mortality in men and women reporters of <1 or 1–2 restaurant prepared meals did not differ from those reporting ≥3 meals/week (P>0.05). The results were robust to effect modification by baseline BMI, years of education, and baseline morbidity. Expectedly, the 24-h dietary intakes of whole grains, fruits, dietary fiber, folate, vitamin C, potassium and magnesium at baseline were lower, but energy, energy density, and energy from fat were higher in more frequent restaurant meal reporters (P<0.05). Baseline serum HDL cholesterol, folate, and some carotenoids were inversely associated with the frequency of eating restaurant prepared meals (P<0.05); however, serum concentrations of total cholesterol, triglycerides, fasting glucose, insulin, glycated hemoglobin, and c-reactive protein were unrelated (P<0.05). The weekly frequency of eating restaurant prepared meals and prospective risk of mortality after 9 years were not related in this cohort.

## Introduction

Eating foods prepared away from home is a popular behavior in the US population [1–3]. In 2005–2008, over a third of the daily energy intake in the US came from foods prepared away from home [3]. Restaurant prepared foods are known to be energy-dense, and higher in fat and sodium, but lower in protective nutrients [3–7]. Expectedly, therefore, frequent consumers of restaurant meals have higher intakes of energy, saturated fat, and sodium, but lower intakes of fruits, vegetables, whole grains, fiber, and several micronutrients [1, 3, 8–10], and lower serum concentrations of vitamins C, E, B-6, folate and carotenoids [2].

Given that the nutritional profiles of restaurant foods are not in accord with foods recommended for risk reduction [11], it is reasonable to posit that frequent consumption of restaurant prepared meals may have adverse health consequences for individuals and contribute to an increased burden of societal health care obligations and expenditures. In some ecologic studies, neighborhoods with greater access and density of fast food restaurants [12, 13] also reported higher prevalence of obesity, but others have reported null findings [14, 15]. Results of some cross-sectional and prospective studies of individual exposure to away from home meals, especially from fast food venues, also suggest an association with body weight [16–18]. Few studies have examined the association of restaurant eating with biomarkers of cardiometabolic risk [2, 19–21]. In the NHANES 2005–2010, we found an inverse association between weekly frequency of eating restaurant meals and HDL cholesterol, but all other examined cardiometabolic biomarkers were unrelated [2]. In an Australian study, weekly frequency of “take-away” meals was unrelated with serum lipid biomarkers in both sexes, but predicted higher serum glucose and insulin in women only [19]. In the CARDIA study cohort, frequency of fast food consumption was related with higher concentrations of cardiometabolic biomarkers at 13–15 years of followup [20, 21].

Associations of neighborhood restaurant density with all-cause mortality and risk of stroke and coronary events have also been reported [22–27]. Available evidence on prospective associations of *individual* exposure to restaurant meals with health outcomes other than BMI is sparse [28–31]. There are reports of increased risk of incident type 2 diabetes [28, 29] and gestational diabetes [30] in association with *individual* exposure to restaurant prepared meals. In the Singapore Chinese Health Study, fast food intake was associated with higher prospective risk of coronary heart disease mortality [31]. To our knowledge, however, there are no published prospective studies of the association of restaurant meal exposure with the risk of cardiometabolic and all-cause mortality in the US population. To fill these gaps, we examined the prospective association of frequency of eating restaurant prepared meals and risk of all-cause and cardiometabolic mortality in a representative sample of the US population. Given some prior reports of adverse cardiometabolic risk biomarker profiles of frequent consumers of restaurant meals, we also examined cross-sectional associations of cardiometabolic biomarkers with frequency of eating away from home meals. Finally, as a measure of validation of the self-reported frequency of eating restaurant meals, we examined biomarkers of dietary exposure and qualitative indicators of dietary intake.

## Methods

We used frequency of eating restaurant prepared meals data from the National Health and Nutrition Examination Surveys (NHANES) conducted in 1999–2000, 2001–2002 and 2003–2004 for this prospective cohort study [32]. The NHANES data have been linked to the national mortality data by the National Center for Health Statistics (NCHS) [33]. The study used anonymized public domain data and was not considered human subjects research by the City University of New York Institutional Review Board. The NHANES are conducted by the NCHS, the Centers for Disease Control and Prevention, to provide information about health status of the US population. The sample design for each NHANES is a stratified, multistage, national probability sample of the non-institutionalized US population [32]. Each survey includes an at-home interview of the sample person and a complete physical examination in a specially equipped mobile examination center (MEC). Dietary intake, anthropometrics, and blood and urine samples are collected in the MEC under standardized conditions. The interview and MEC response rates for the surveys in the study were >70% [34].

The surveys selected (1999–2000, 2001–2002, 2003–2004) for the current study were determined by the availability of cycles where similar question about eating restaurant prepared meals was asked to elicit this information. Surveys conducted prior to 1999–2000 did not query this information. In surveys conducted after 2004, the question used to obtain restaurant meal frequency changed, and was not comparable to earlier surveys.

### Exposure assessment

The weekly frequency of eating restaurant prepared meals was the principal exposure in this study and was operationalized from a question asked during the household interview: “On average, how many times per week do you eat meals that were prepared in a restaurant? Please include eat-in restaurants, carry-out restaurants, and restaurants that deliver food to your house.” [32]. Analyses were conducted using weekly frequency categories of eating restaurant prepared meals of <1, 1–2 or ≥3 times/week and also using the median frequency for the three categories of frequency as a test for trend.

### Outcome ascertainment

#### All-cause mortality outcome

Adult respondents to the NHANES 1999–2004 have been linked to the National Death Index (NDI) with the latest available follow-up to Dec. 31, 2011 [35]. The NCHS determined the mortality status of NHANES respondents primarily through probabilistic matching to the NDI with supplemental information from the Social Security Administration, the Centers for Medicare and Medicaid Services, and death certificates [35]. In a validation study, the NCHS applied the NHANES NDI matching algorithm to the NHANES I Epidemiologic Followup Study cohort with known mortality status. The NCHS matching procedures correctly classified 98% of subjects [36].

To preserve subject anonymity, the public domain data on personal identifiers (but not vital status) were subjected to perturbation techniques by the NCHS [33]. The actual date of death is not available in the public-use mortality linked file. Instead, NCHS computed the person months of followup from the date of the household interview or the date of the MEC visit. The public domain mortality files only include information on mortality from nine leading causes of death and a residual category of all other causes based on the Tenth International Classification of Diseases (ICD-10) codes for underlying cause of death and comprised the all-cause mortality cases in our analysis [33].

#### Cause-specific outcome

Given the published reports of increased cardiometabolic risk in relation to away from home eating, we created a cause-specific outcome (cardiometabolic diseases) by combining deaths due to diseases of the heart (ICD-10 codes I00-I09, I11, I13, I20-I51), cerebrovascular diseases (ICD-10 codes I60-I69), and diabetes mellitus (ICD-10 codes E10-E14), as available in the public domain file [33].

#### Biomarker concentrations at baseline

We also examined a number of biomarkers, measured at baseline, in relation to frequency of eating restaurant prepared meals. The nutritional and metabolic biomarker concentrations were assayed in blood samples collected in the MEC using standardized collection and assay protocols [32]. The cardiometabolic biomarkers examined included serum concentrations of total and HDL cholesterol, fasting triglycerides, fasting glucose, fasting insulin, fasting c-peptide, glycated hemoglobin, and c-reactive protein. These cardiometabolic biomarkers have been examined in prior studies in relation to frequency of restaurant eating [2, 19–21]. The nutritional biomarkers examined included serum and red blood cell folate, vitamins C, D and E, and carotenoids. These biomarkers (except serum vitamin D) are good indicators of dietary exposure of the relevant nutrient for which fruits and vegetables are good sources. Serum vitamin D is the recommended indicator of vitamin D nutritional status and reflects both dietary exposure and cutaneous synthesis [37].

#### Dietary intakes at baseline

The NHANES documentation provides no information on validation of the question used to determine weekly frequency of eating restaurant prepared meals. To provide some degree of validation for this measure, we examined the associations of categories of eating restaurant prepared meals with self-reported dietary intakes measured at baseline. The dietary intakes were estimated from a 24-hour dietary recall obtained at the time of the MEC interview [32]. These included intakes of energy, energy density of foods (kcal/g), percent energy from macronutrients, and energy-adjusted dietary fiber, vitamins C and E, folate, sodium, potassium, magnesium, and serving equivalents of fruits, vegetables, whole grains, added sugar, and discretionary fat.

### Analytic sample

All respondents aged ≥40 y at baseline in 1999–2004 were eligible for inclusion in the study (n = 9970). We excluded pregnant and lactating women (n = 11), respondents missing the MEC exam (n = 825), missing information on the weekly frequency of eating restaurant prepared meals (n = 6), missing mortality follow up due to lack of NDI matching (n = 14), and missing followup time (n = 7), for a final analytic sample of 9107 respondents.

### Covariates for analysis

Variables with associations with our exposure and outcome, i.e., potential confounders, were available for each survey. Covariates were chosen based on established correlates of dietary exposure and mortality. These included age, race/ethnicity, family poverty income ratio, years of formal education, self-reported tobacco exposure, alcohol use, measured body mass index, any leisure-time physical activity, and self-report of whether a doctor had informed the respondent of a diagnosis of heart disease, stroke, diabetes, angina, heart attack, congestive heart failure, hypertension, cancer, and emphysema.

### Statistical analysis

Person time of follow-up was computed from the date of the NHANES examination to the last date known alive or the end date of followup (12/31/2011), which ever came first. We describe the socio-demographic and life-style characteristics of respondents in categories of weekly frequency of eating restaurant prepared meals. We used survey sample-weighted Cox proportional hazards regression models to estimate the hazard ratio of all-cause or cardiometabolic mortality in relation to weekly frequency of eating restaurant prepared meals. In these analyses, attained age during followup was the underlying time metric [38]. These models were fit separately for men and women and included the above mentioned multiple potential confounders of the eating out and mortality association. The tables present the covariate-adjusted hazard ratio and 95% CI of mortality in categories of eating restaurant prepared meals, with the highest category (≥3meals/week) as the reference category. The P-values based on a Wald’s F statistic for the test of hazard of mortality with eating restaurant prepared meals as a trend variable are presented [38]. We excluded from regression models, respondents missing information on education (n = 25), smoking status (n = 15), and leisure time physical activity (n = 1) due to small numbers. Larger numbers of respondents were missing information on poverty income ratio (n = 854), measured BMI (n = 384), and alcohol drinking status (n = 602), and were retained as unknown category for each variable in regression analyses.

### Sensitivity analysis

Both energy intake and BMI may mediate the association of eating restaurant prepared meals with health outcomes [12, 28, 29]. Therefore, we also examined the eating out and mortality associations without adjustment for baseline BMI or 24-h energy intake. Those with undiagnosed pre-clinical disease may have lower frequency of eating restaurant prepared meals at baseline, but may have higher subsequent mortality risk due to disease progression. To exclude this possibility of reverse causation, we examined the association of mortality and restaurant meal frequency after exclusion of events that occurred in the first 2 years of follow up. Similarly, to exclude reverse causation due to lower frequency of eating restaurant prepared meals due to *known* disease at baseline, which may also relate to higher subsequent mortality, we examined the associations of interest in analyses stratified by self-reported medical condition status at baseline.

We also reexamined the above associations after exclusion of accidental and unknown causes of mortality. Finally, to examine whether the association of restaurant eating with the risk of all-cause or cardiometabolic mortality was modified by key covariates, we tested the interaction of frequency of eating restaurant meal with baseline BMI, morbidity, and years of formal education.

We assessed the proportionality of the hazards obtained from Cox regressions by forming three attained age categories of approximately the same number of deaths and tested the statistical significance of the interaction between these categories and the categories of the frequency of eating meals away from home using a Wald test. A similar assessment of proportionality of the hazards for the Cox regressions with eating restaurant prepared meals as a trend variable was conducted by testing the interaction between the 3 age dependent categories and the trend variable [38].

### Associations of biomarkers and dietary intakes at baseline with frequency of eating restaurant meals

We used multiple-covariate adjusted linear regression methods to examine the cross-sectional association of frequency of eating restaurant meals with biomarkers and dietary characteristics. The confounders for biomarker outcomes in these models included baseline age, sex, race/ethnicity, education, income, month of MEC exam, hours of fasting before phlebotomy, supplement use, history of chronic disease, serum cotinine (marker of nicotine exposure), BMI, leisure time physical activity, and alcohol use. Models to examine the self-reported dietary nutrient and food group outcomes were adjusted for baseline age, sex, race/ethnicity, income, education, BMI, weekday of recall intake, month of recall, and self-reported chronic disease status. The tables present predicted margins (i.e., adjusted means) and 95% CIs from multiple-covariate adjusted linear regression models [39].

We used SAS 9.2 (SAS Institute, Cary, NC) and SAS callable SUDAAN 11.0.0 [40] to account for sample weighting and other aspects of the complex sample design of the NHANES [41]. All P-values are two-sided and are not corrected for multiple comparisons.

## Results

### Socio-demographic and lifestyle characteristics of respondents by weekly frequency of eating restaurant prepared meals

Slightly over 10% of all respondents reported that they never ate restaurant meals, but over a third reported eating ≥3 restaurant prepared meals/week (Table 1). Reports of 3 or more restaurant prepared meals/week were more prevalent among men, non-Hispanic whites, 40–59 year olds, those with higher BMI, higher income and education, current drinkers, those who reported some leisure time physical activity, and no chronic disease (P<0.0001) (Table 1). Overall, men and women reported a mean of 2.75 and 2.15 restaurant prepared meals/week, respectively (online supplemental Table 1).

**Table 1:** Cohort characteristics (weighted % and 95% CI) by weekly frequency of eating restaurant prepared meals^1^: US adults, aged ≥ 40 y, NHANES 1999–2004.

		Weekly frequency of eating restaurant prepared meals		
	All	<1 time	1–2 times	≥3 times
All (n)	9107	3248 (27.4%)	3428 (38.6%)	2431 (34.0%)
Years of follow-up (median)	9.2	9.2	9.2	9.3
% Women	52.7 (51.7, 53.7)	58.5 (56.6, 60.3)	54.9 (53.2, 56.7)	45.6 (42.8, 48.3)
**Race/ethnicity**				
Non-Hispanic white, n = 4870	77.1 (73.5, 80.3)	65.3 (59.4, 70.7)	78.8 (74.8, 82.2)	84.8 (82.3, 87.0)
Non-Hispanic Black, n = 1739	10.0 (8.2, 12.2)	15.5 (12.6, 18.9)	8.8 (7.0, 11.0)	6.9 (5.5, 8.6)
Mexican-American, N = 1918	4.6 (3.3, 6.4)	5.8 (3.9, 8.5)	5.1 (3.5, 7.3)	3.1 (2.3, 4.2)
Other, n = 580	8.3 (6.2, 11.0)	13.4 (9.9, 18.0)	7.4 (5.3, 10.1)	5.1 (3.6, 7.3)
**Age, y**				
40–59 n = 4139	63.2 (61.7, 64.7)	51.6 (48.6, 54.6)	62.6 (60.5, 64.7)	73.1 (70.4, 75.6)
≥60 n = 4968	36.8 (35.3, 38.3)	48.4 (45.4, 51.3)	37.3 (35.3, 39.5)	26.9 (24.4, 29.6)
**Poverty Income Ratio, %**				
<130, n = 2262	17.1 (15.1, 19.3)	29.7 (26.3, 33.3)	15.4 (13.2, 17.9)	8.9 (7.4, 10.6)
130–349, n = 3201	31.7 (29.9, 33.5)	36.9 (34.1, 39.7)	32.8 (30.3, 35.3)	26.3 (24.1, 28.7)
≥350, n = 2790	43.1 (40.5, 45.8)	24.0 (21.0, 27.2)	44.5 (41.0, 48.0)	57.1 (54.3, 59.8)
Unknown, n = 854	8.0 (6.8, 9.4)	9.5 (7.6, 11.8)	7.4 (6.0, 8.9)	7.7 (6.0, 9.8)
**Education, y**				
<12, n = 3297	21.9 (20.2, 23.8)	35.9 (32.8, 39.2)	19.4 (17.6, 21.3)	13.6 (11.8, 15.6)
12, n = 2094	26.1 (24.6, 27.7)	24.8 (22.7, 27.1)	28.7 (26.2, 31.3)	24.3 (22.2, 26.5)
Some College, n = 2101	27.7 (26.3, 29.2)	23.7 (21.3, 26.2)	28.5 (26.3, 30.7)	30.1 (27.9, 32.4)
>college, n = 1590	24.2 (21.9, 26.6)	15.5 (13.4, 18.0)	23.4 (20.6, 26.6)	32.0 (28.6, 35.6)
**Smoking Status**				
Never, n = 4275	46.7 (45.0, 48.4)	44.9 (41.8, 48.0)	46.9 (44.6, 49.2)	48.0 (45.2, 50.8)
Former, n = 3078	32.6 (31.2, 34.1)	31.4 (29.4, 33.5)	33.7 (31.0, 36.5)	32.4 (30.2, 34.6)
Current, n = 1739	20.6 (19.3, 22.0)	23.6 (21.3, 26.2)	19.4 (17.3, 21.6)	19.6 (17.7, 21.7)
**Alcohol Drinking status**				
Never, n = 1326	12.4 (10.7, 14.3)	16.8 (14.4, 19.6)	11.8 (9.5, 14.6)	9.4 (7.7, 11.4)
Former, n = 1594	17.0 (15.7, 18.5)	18.9 (17.2, 20.8)	17.8 (15.9, 19.8)	14.7 (12.7, 16.9)
Current, n = 5585	64.8 (62.2, 67.3)	56.5 (53.0, 59.8)	65.6 (62.0, 69.0)	70.6 (67.7, 73.4)
Unknown, n = 602	5.8 (5.2, 6.5)	7.8 (6.5, 9.3)	4.8 (4.0, 5.7)	5.3 (4.5, 6.4)
**Body mass index, kg/m**^**2**^				
<25.0, n = 2452	28.6 (26.8, 30.5)	31.3 (29.2, 33.6)	29.1 (26.4, 32.0)	25.8 (22.9, 29.0)
25.0–29.9, n = 3339	35.9 (34.5, 37.2)	34.0 (31.8, 36.2)	35.9 (33.9, 38.0)	37.3 (35.2, 39.5)
≥30, n = 2932	32.4 (30.7, 34.3)	29.8 (27.4, 32.3)	32.3 (29.8, 35.0)	34.7 (32.0, 37.6)
Unknown, n = 384	3.1 (2.5, 3.8)	4.9 (3.8, 6.3)	2.6 (1.9, 3.6)	2.1 (1.5, 2.9)
**Any leisure physical activity**				
Yes, n = 4853	61.6 (59.3, 63.8)	51.8 (48.0, 54.9)	62.4 (59.6, 65.1)	68.8 (65.7, 71.7)
None, n = 4253	38.4 (36.2, 40.7)	48.5 (45.1, 52.0)	37.6 (34.9, 40.4)	31.2 (28.3, 34.3)
**Self-reported chronic disease**				
Yes, n = 5221	50.6 (48.3, 52.8)	55.5 (52.5, 58.5)	51.7 (48.6, 54.7)	45.4 (42.2, 48.6)
No, n = 3886	49.4 (47.1, 51.7)	44.5 (41.5, 47.5)	48.3 (45.3, 51.4)	54.6 (51.4, 57.8)

^1^All variables listed in the table were significantly associated with weekly frequency of eating restaurant prepared meals (Chi square test of independence for all variables except smoking status was P<0.0001 (for smoking status the P = 0.03)). Respondents with unknown information on education (n = 25), smoking status (n = 15), and any leisure time physical activity (n = 1) were excluded.

### Weekly frequency of eating restaurant prepared meals and risk of mortality

In this cohort, mean age 56.7±0.22 years at baseline, 1226 men and 974 women died from all-causes over a median follow up period of 9.2 years. Of the total, 373 men and 292 women died from cardiometabolic causes. In Cox proportional hazards regression models adjusted for potential confounders, the hazard ratio of mortality from all-causes or from cardiometabolic diseases did not differ by frequency of eating restaurant prepared meals in men (Table 2) or women (Table 3). In these analyses, the confidence intervals for hazard ratios were wide, included one, and tests of trend across categories of eating restaurant meals/week were not significant (P>0.05). The observed lack of an association between frequency of eating restaurant prepared meals and mortality was essentially unchanged in models that included energy intake or excluded BMI as an explanatory variable (Tables 2 and 3).

**Table 2:** ^1^Covariate-adjusted hazard ratio of mortality from all-causes and cardiometabolic^2^ causes in relation to weekly frequency of eating restaurant prepared meals after 9 years of follow-up: US men, aged ≥ 40 y at baseline.

	Weekly frequency of eating restaurant prepared meals			P^3^
	<1	1–2	≥3 (reference)	
**All-cause mortality**				
Model 1^4^ N = 4478; events = 1214	0.86 (0.70, 1.06)	0.88 (0.68, 1.14)	1.0	0.2
Model 2^5^ N = 4260; events = 1129	0.87 (0.70, 1.07)	0.88 (0.68, 1.14)	1.0	0.2
Model 3^6^ N = 4478; events = 1214	0.88 (0.72, 1.08)	0.87 (0.68, 1.13)	1.0	0.2
**Cardiometabolic mortality as the underlying cause**				
Model 1^4^ N = 4478; events = 370	1.33 (0.95, 1.85)	0.93 (0.63, 1.37)	1.0	0.4
Model 2^5^ N = 4260; events = 339	1.29 (0.89, 1.86)	0.89 (0.62, 1.29)	1.0	0.6
Model 3^6^ N = 4478; events = 370	1.37 (0.99, 1.88)	0.93 (0.63, 1.37)	1.0	0.3

^1^Estimates are hazard ratios and 95% CIs from Cox proportional hazards regression models.

^2^Cardiometabolic causes included cardiovascular and diabetes causes.

^3^P value associated with weekly frequency of eating restaurant prepared meals as a trend.

^4^Model 1: Independent variables included: number of times/week eat restaurant prepared meals (<1, 1–2, ≥3), race/ethnicity (non-Hispanic White, non-Hispanic Black, Mexican-American, Other), poverty income ratio, % (<130, 130–349, ≥350, unknown), education, y (<12, 12, some college, >college), body mass index, kg/^m2^ (<25, 25–29.9, ≥30, unknown), smoking status (current smoker, former smoker, never smoked), alcohol drinking status (current drinker, former drinker, never drank, unknown), self-reported doctor diagnosed chronic disease (yes, no), any leisure-time physical activity (yes, no).

^5^Model 2: Included energy intake, kcal, (continuous) in model 1.

^6^Model 3: excluded BMI from model 1.

**Table 3:** ^1^Covariate-adjusted hazard ratio of mortality from all-causes and cardiometabolic^2^ causes in relation to weekly frequency of eating restaurant prepared meals after 9 years of follow-up: US women aged ≥ 40 y at baseline.

	Weekly frequency of eating restaurant prepared meals			P^3^
	<1	1–2	≥3 (reference)	
**All-cause mortality**				
Model 1^4^ N = 4591; events 968	0.89 (0.67, 1.17)	0.94 (0.71, 1.24)	1.0	0.4
Model 2^5^ N = 4368; events = 907	0.89 (0.67, 1.18)	0.94 (0.71, 1.24)	1.0	0.5
Model 3^6^ N = 4591; events 968	0.89 (0.67, 1.17)	0.94 (0.71, 1.24)	1.0	0.5
**Cardiometabolic causes as the underlying cause**				
Model 1^4^ N = 4591; events = 291	0.75 (0.45, 1.25)	0.84 (0.52, 1.36)	1.0	0.3
Model 2^5^ N = 4368; events = 268	0.73 (0.44, 1.23)	0.83 (0.51, 1.35)	1.0	0.2
Model 3^6^ N = 4591; events = 291	0.75 (0.46, 1.24)	0.85 (0.52, 1.38)	1.0	0.3

^1^Estimates are hazard ratios and 95% CIs from Cox proportional hazards regression models.

^2^Cardiometabolic causes included cardiovascular and diabetes.

^3^P value associated with weekly frequency of eating restaurant prepared meals as a trend.

^4^Model 1: Independent variables included: number of times/week eat restaurant prepared meals (<1, 1–2, ≥3), race/ethnicity (non-Hispanic White, non-Hispanic Black, Mexican-American, Other), poverty income ratio, % (<130, 130–349, ≥350, unknown), education, y (<12, 12, some college, >college), body mass index, kg/m^2^ (<25, 25–29.9, ≥30, unknown), smoking status (current smoker, former smoker, never smoked), alcohol drinking status (current drinker, former drinker, never drank, unknown), self-reported doctor diagnosed chronic disease (yes, no), any leisure-time physical activity (yes, no),

^5^Model 2: Included energy intake, kcal, (continuous) in model 1.

^6^Model 3: excluded BMI from model 1.

### Sensitivity analyses

Results were unchanged in models with adjustment for history of post-menopausal hormone use in women, exclusion of first 2-years of follow-up, exclusion of deaths due to accident or unknown cause of death, or stratification for self-reported history of known medical conditions at baseline (P>0.05) (Online Supplemental Tables 2 and 3). Similarly, all associations were null in models that tested for the interaction of frequency of eating restaurant meals with baseline BMI, self-reported chronic disease, and education, for predicting the hazard of mortality from all causes or cardiometabolic diseases (P>0.05).

Tests for lack of proportionality of hazard for men and women were not significant for mortality from cardiometabolic causes, and for men, were not statistically significant for all-cause mortality but were significant (P = 0.006) for women. Because the hazard ratios for all-cause mortality among women were similar across the three attained age categories (i.e., quantitative interactions), we only present the overall hazard ratios in Table 3.

### Associations of weekly frequency of eating restaurant prepared meals with cardiometabolic and nutritional biomarkers

In covariate-adjusted models, the serum HDL-cholesterol concentration decreased with increasing frequency of eating restaurant prepared meals (P = 0.03) (Table 4). All other metabolic biomarkers examined (total cholesterol, c-reactive protein, and fasting triglycerides, glucose, and insulin, glycated hemoglobin and c-peptide) were unrelated with frequency of eating restaurant prepared meals (P>0.05). With increasing frequency of eating restaurant prepared meals, concentrations of serum and RBC folate, and serum α-carotene, β-carotene, and β-cryptoxanthin decreased (P<0.05), serum lycopene increased (P<0.0001), but serum vitamins C, D, and E were unrelated (P>0.05) (Table 4).

**Table 4:** ^1^Covariate-adjusted geometric mean and 95% CI of serum concentration of cardiometabolic and nutritional biomarkers measured at baseline, by weekly frequency of eating restaurant prepared meals: US adults, aged ≥ 40, NHANES 1999–2004.

Serum Biomarker	Weekly frequency of eating restaurant prepared meals			
	<1	1–2	≥3	P^2^
**Cardiometabolic biomarkers**				
Total Cholesterol, mg/dl n = 8423	212 (210, 215)	213 (211, 215)	212 (209, 215)	0.6
HDL cholesterol, mg/dl n = 8411	53.5 (52.5, 54.4)	53.2 (52.3, 54.1)	52.4 (51.6, 53.2)	0.03
Triglycerides^3^, mg/dl n = 3767	133 (127, 139)	135 (129, 140)	132 (127, 138)	0.6
Glucose^3^, mmol/L n = 3780	5.76 (5.66, 5.87)	5.65 (5.58, 5.72)	5.73 (5.63, 5.84)	0.5
Insulin^3^, uU/ml n = 3764	55.2 (51.9, 58.6)	55.8 (53.3, 58.5)	56.2 (53.1, 59.5)	0.7
C-peptide^3^, nmol/L n = 3672	0.76 (0.72, 0.79)	0.77 (0.75, 0.80)	0.78 (0.76, 0.81)	0.2
Glycated hemoglobin, % n = 8334	5.58 (5.54, 5.63)	5.57 (5.52, 5.62)	5.58 (5.53, 5.62)	0.9
C-reactive protein, mg/dl n = 8438	0.22 (0.20, 0.24)	0.23 (0.21, 0.24)	0.23 (0.22, 0.24)	0.4
**Nutritional biomarkers**				
Folate, nmol/L N = 8427	45.3 (44.2, 46.4)	45.6 (44.0, 47.1)	43.9 (42.7, 45.1)	0.02
RBC folate, nmol/L N = 8382	1271 (1244, 1299)	1262 (1232, 1292)	1239 (1208, 1272)	0.04
Vitamin C^4^, umol/L N = 2871	57.4 (53.5, 61.3)	56.4 (53.3, 59.5)	56.0 (52.9, 59.0)	0.5
Vitamin D^5^, nmol/L N = 5823	58.2 (55.8, 60.6)	57.8 (55.6, 60.1)	57.5 (55.3, 59.8)	0.5
Vitamin E, umol/L N = 8374	32.5 (31.8, 33.3)	32.5 (31.7, 33.4)	32.2 (31.5, 33.0)	0.4
α-carotene^5^, umol/L n = 5830	0.056 (0.049, 0.065)	0.053 (0.048, 0.059)	0.050 (0.046, 0.054)	0.004
β-carotene^5^, umol/L n = 5830	0.29 (0.26, 0.31)	0.27 (0.25, 0.28)	0.26 (0.24, 0.28)	0.009
Lutein+zeaxanthin^5^, umol/L n = 5827	0.26 (0.25, 0.28)	0.25 (0.24, 0.26)	0.25 (0.24, 0.26)	0.07
β-cryptoxanthin^5^, umol/l n = 5815	0.13 (0.12, 0.14)	0.13 (0.12, 0.13)	0.12 (0.11, 0.13)	0.003
Lycopene^5^, umol/l n = 5827	0.31 (0.30, 0.33)	0.34 (0.33, 0.35)	0.36 (0.35, 0.37)	0.0008

^1^Estimates are predicted margins (adjusted means) and 95% CIs from linear regression models with each biomarker as a continuous outcome. Independent variables included weekly frequency of eating restaurant prepared (<1, 1–2, ≥3), sex, age, y (40–59, ≥60), race/ethnicity (non-Hispanic White, non-Hispanic Black, Mexican-American, Other), poverty income ratio, % (<130, 130–349, ≥350, unknown), education, y (<12, 12, some college, >college), body mass index, kg/m^2^ (<25, 25–29.9, ≥30, unknown), alcohol drinking status (current drinker, former drinker, never drank, unknown) serum cotinine (continuous), any supplement use (yes, no), month of MEC exam (November-April, May-October)), hours of fasting before phlebotomy (continuous), self-reported doctor diagnosed chronic disease (yes, no), and any leisure-time physical activity (yes, no). n refers to number of observations used in the regression model for each biomarker.

All biomarkers except serum total and HDL cholesterol, and vitamin C were log transformed.

For vitamin E and carotenoids, serum cholesterol was an additional covariate.

^2^P value associated with weekly frequency of eating restaurant prepared meals as a trend.

^3^Fasting subsample.

^4^Measured in 2003–2004 only.

^5^Measured in 2001–2002 and 2003–2004 only.

### Associations of weekly frequency of eating restaurant prepared meals and qualitative dietary characteristics of 24-dietary intake

In multiple linear regression analyses, the 24-h intakes of energy, energy density of foods, and % energy from fat increased with increasing weekly frequency of eating restaurant prepared meals (P<0.05) (Table 5). The intakes of % energy from carbohydrates, and energy-adjusted fiber, vitamin C, folate, potassium, magnesium, servings of fruits, and whole grains decreased with increasing frequency of restaurant meals (P<0.05). Intakes of energy from protein, vitamin E, sodium intake, added sugar, and discretionary solid fat were unrelated with frequency of restaurant meal consumption (P>0.05).

**Table 5:** Adjusted mean and 95% CI^1^ of self-reported 24-h dietary nutrient and food group intake by weekly frequency of eating restaurant prepared meals: US adults, aged > = 40, NHANES 1999–2004.

	Weekly frequency of eating restaurant prepared meals			P^2^
	<1	1–2	≥3	
Energy, Kcal	1973 (1917, 2030)	2001 (1962, 2039)	2128 (2086, 2170)	<0.0001
Energy density of foods only (kcal/g) (n = 8636)	1.80 (1.76, 1.84)	1.88 (1.85, 1.91)	1.95 (1.90, 1.99)	0.0001
Energy from saturated fat, %	10.6 (10.4, 10.9)	10.9 (10.7, 11.1)	11.0 (10.7, 11.4)	0.08
Energy from protein, %	15.8 (15.4, 16.1)	15.5 (15.2, 15.7)	15.4 (15.2, 15.7)	0.3
Energy from carbohydrates, %	50.4 (49.6, 51.1)	49.7 (49.2, 50.2)	48.5 (47.8, 49.3)	0.0005
Fiber/100 g of carbohydrates	7.01 (6.8, 7.2)	6.75 (6.6, 6.9)	6.5 (6.3, 6.7)	0.005
Fiber^3^, g	16.9 (16.3, 17.5)	15.9 (15.4, 16.4)	15.3 (14.6, 16.0)	0.0005
Vitamin C^3^, mg	101 (92, 109)	91 (87, 96)	88 (80, 95)	0.04
Vitamin E^3^, mg (α-tocopherol)	7.8 (7.4, 8.2)	7.6 (7.3, 7.8)	7.6 (7.2, 8.0)	0.9
Folate^3^, ug	399 (383, 410)	386 (376, 397)	376 (362, 391)	0.03
Vitamin B-6^3^, mg	1.86 (1.80, 1.92)	1.79 (1.74, 1.84)	1.77 (1.71, 1.84)	0.1
Sodium^3^, mg	3209 (3124, 3295)	3247 (3174, 3319)	3264 (3172, 3356)	0.5
Potassium^3^, mg	2856 (2770, 2941)	2732 (2671, 2794)	2696 (2627, 2764)	0.01
Magnesium^3^, mg	291 (284, 299)	275 (268, 282)	274 (266, 281)	0.01
Fruit^3^, cup equivalents, cups	1.25 (1.13, 1.37)	1.14 (1.07, 1.21)	1.0 (0.9, 1.1)	0.0004
Vegetable^3^, cup equivalents	1.71 (1.62, 1.8)	1.60 (1.54, 1.66)	1.63 (1.55, 1.71)	0.6
Whole grain^3^, oz equivalents	0.83 (0.76, 0.91)	0.70 (0.65, 0.75)	0.66 (0.59, 0.73)	0.01
Added sugar^3^, tsp	17.0 (16.1, 18.0)	17.8 (17.0, 18.6)	18.3 (17.4, 19.2)	0.09
Discretionary solid fat^3^, g	43.6 (42.6, 44.6)	44.5 (43.3, 45.7)	45.3 (44.0, 46.7)	0.06

^1^Estimates are predicted margins (adjusted means) and 95% CIs from linear regression models with each dietary variable as a continuous outcome. Independent variables included, weekly frequency of eating restaurant prepared (<1, 1–2, ≥3), sex, age, y (40–59, ≥60), race/ethnicity (non-Hispanic White, non-Hispanic Black, Mexican-American, Other), poverty income ratio, % (<130, 130–349, ≥350, unknown), education, y (<12, 12, some college, >college), body mass index, kg/^m2^ (<25, 25–29.9, ≥30, unknown), weekday of recalled intake (Monday-Thursday, Friday-Sunday), month of MEC exam (November to April, May-October), and self-reported doctor diagnosed chronic disease (yes, no).

N = 8645 (respondents with information on all covariates in the model), except where n is included in the table.

^2^P value associated with weekly frequency of eating restaurant prepared meals as a trend.

^3^Energy-adjusted models included total energy intake, kcals (continuous).

## Discussion

In this national cohort of middle-aged men and women, the risks of mortality from all-causes or cardiometabolic diseases and frequency of eating restaurant prepared meals were unrelated after a median 9 years of follow-up. To our knowledge, no published studies have examined the specific study question to allow corroboration. Apart from body weight as a prospective outcome of mostly fast food exposure [17, 18], only a few prospective studies have examined the risk of specific chronic disease or mortality outcomes in relation to eating restaurant prepared meals [28–31]. The 10-y risk of type 2 diabetes increased in relation to frequency of consuming selected restaurant food items [28], but decreased in association with the number of midday or evening meals prepared at home [29]. In a Spanish cohort, gestational diabetes risk was higher in women who reported ≥2 servings/week of fast foods, defined as hamburger, sausage, and pizza [30]. In a Singapore Chinese cohort, frequent consumption of selected Western-style fast food items increased the risk of CHD mortality and incident type 2 diabetes [31]. Notably, the exposure in these studies were selected meals [29], fast food or “western” items [28, 30, 31], and were not directly comparable to ours.

The reasons for the null findings of our study are not known, but we can speculate about some possibilities. Consumption of foods prepared in restaurants may possibly increase health risk due to higher energy content and poor nutritional quality of the offerings. Although fast foods have been the focus of past studies [28, 30, 31], it is noteworthy that content of foods on menus at all types of restaurants were found to be high in energy, saturated fat, and sodium [4–6, 42]. In a comprehensive analysis, the energy content and nutritional profiles of offerings in popular full-serve chain and non-chain restaurants were *worse* relative to fast food outlets [4, 5]. Moreover, in the US population, self-reported nutrient intakes of consumers of both restaurant and fast food meals were comparably inferior to those reporting home-prepared meals [43]. We also found that the associations of metabolic or nutritional biomarkers with meals prepared away from home in all restaurants or only fast food venues were similar [2]. Therefore, the use of all restaurant prepared meals, rather than fast foods only as the exposure, is an unlikely cause of null findings in our study.

Classification into exposure categories of weekly frequency of eating restaurant prepared meals is dependent on the validity of the question used to elicit this information. The NHANES documentation, however, provides no information on validation of this question. In the absence of known biomarkers of consumption of restaurant prepared meals, we examined both objective (nutritional biomarkers) and subjective (self-reported nutrient and food group intakes) indicators of dietary exposure by categories of eating restaurant prepared meals used in our analyses. The directions of several key nutritional biomarkers and of self-reported intakes of energy, energy density, key food groups, and nutrients estimated from a 24-hour recall were consistent with the expected relationship with frequency of eating restaurant prepared meals. Although these associations suggest that the question about eating restaurant prepared may have been somewhat successful in this regard, we cannot exclude the possibility of misclassification into exposure categories due to misreporting of frequency of restaurant meal consumption.

It is also possible that food selections at home or away of those who report more frequent consumption of restaurant prepared meals may differ from those who eat out less frequently. An occasional restaurant prepared meal may possibly include more indulgent food selections. On the other hand, frequent consumers of restaurant meals may make selections that are more judicious at home and away. However, to our knowledge, this question remains to be explored in adults. In children who do not usually control food availability at meals, fast food meal consumers also had poor quality of home foods [44]. Moreover, in the current study, the inverse associations of frequency of restaurant meal consumption with at-risk food groups and serum concentrations of folate and three carotenoids counter the likelihood of more careful food selection by frequent eaters of restaurant meals.

Because of reports of higher BMI of frequent consumers of restaurant meals, it has been suggested that BMI may mediate the relation of restaurant foods with health outcomes such as metabolic biomarkers and chronic disease such as diabetes and heart disease [12, 28, 29]. Accordingly, we also examined all such associations after exclusion of baseline BMI from regression models, yet the findings were essentially unchanged. We note that similar to our previous study [2], in the current study, we found no cross-sectional associations of reported frequency of eating restaurant prepared meals with cardiometabolic biomarkers at study baseline. Interestingly, in two studies that examined the acute effect of feeding fast food meals under controlled conditions, vascular function and cardiometabolic biomarkers did not differ after fast-food and healthier alternative meals [45, 46].

Strengths of our study include a nationally representative cohort with availability of measured baseline BMI and multiple putative confounders, and restaurant exposure assessment from a frequency question. We could assess the validity of this question to some extent against both self-reported dietary intake in a 24-h recall and objective nutritional biomarkers. Results of sensitivity analyses suggest no effect modification due to baseline morbidity, education, and BMI.

Although our analytic approach adjusted for other putative confounders of the mortality association with eating of restaurant prepared meals, we acknowledge the possibility of residual confounding due to poorly measured or unknown confounders as this is an observational study. We also note that there is no repeat assessment of the exposure or other correlates of mortality over the period of followup in the NHANES. For example, both the frequency and types of restaurant and physical activity exposures may change over time and contribute to null findings observed in the study. Finally, given the latency of most metabolic conditions, associations may emerge after a longer period of followup than available in the current study.

The results of this study should not be interpreted as an endorsement of frequent consumption of restaurant prepared meals or of the food selections available in US restaurants. Individuals and families choose to consume away from home meals for a variety of reasons that range from convenience to socialization. Despite expressed concerns about what is on the menu at US restaurants [47, 48], assessment of trends in changes in quality of offerings at fast food or other traditional restaurants suggests only modest progress [7, 49–51]. In addition, consumers do not often select the so-called healthy offerings available on the menu [52]. On the other hand, home cooking allows control on types of foods, methods of preparation, and the quantities in which they are served, and may help in attaining recommended dietary patterns [3, 53, 54]. However, despite the popularity of cooking shows, cooking in American homes is continuing to decline [55].

## Conclusions

Although both self- reported food group and nutrient intakes, and serum concentrations of several nutritional biomarkers in more frequent reporters of restaurant meals were suggestive of poor quality of diets reported at baseline, we found no prospective associations with mortality from all-causes or cardiometabolic diseases in this national cohort.

## Supporting information

S1 Table Covariate-adjusted mean weekly frequency of eating restaurant prepared meals, by sex, by socio-demographic characteristics of the analytic sample. (DOCX)

S2 Table Sensitivity analysis: Covariate-adjusted hazard ratio of mortality from all-causes and cardiometabolic diseases in relation to weekly frequency of eating restaurant prepared meals after 9 years of follow-up: US men, aged ≥40 y at baseline. (DOCX)

S3 Table Sensitivity analysis: Covariate-adjusted hazard ratio of mortality from all-causes and cardiometabolic diseases in relation to weekly frequency of eating restaurant prepared meals after 9 years of follow-up, US women, aged ≥40 y at baseline. (DOCX)
