# Supplementary material for: A prospective study of frequency of eating restaurant prepared meals and subsequent 9-year risk of all-cause and cardiometabolic mortality in US adults
Source: PLoS One. 2018 Jan 23;13(1):e0191584. doi: 10.1371/journal.pone.0191584 (PMC5779659; doi:10.1371/journal.pone.0191584)
Supplement: S1 Table — (DOCX) [file pone.0191584.s001.docx]

**Supporting Information**

**S1 Table. ^1^Covariate-adjusted mean weekly frequency of eating restaurant prepared meals, by sex, by socio-demographic characteristics of the analytic sample**

|  | **Men (n=4478)** | | **Women (n=4591)** | |
| --- | --- | --- | --- | --- |
|  | Mean (95% CI) | P value of Wald’s F test | Mean (95% CI) | P value of Wald’s F test |
| **All** | 2.75 (2.60, 2.89) |  | 2.15 (2.01, 2.29) |  |
| **Race/ethnicity** |  | <0.0001 |  | 0.0004 |
| Non-Hispanic white | 3.00 (2.79, 3.21) |  | 2.21 (2.04, 2.37) |  |
| Non-Hispanic Black | 2.05 (1.86, 2.23) |  | 1.58 (1.43, 1.72) |  |
| Mexican-American | 2.32 (2.12, 2.52) |  | 1.80 (1.57, 2.03) |  |
| Other | 2.23 (1.84, 2.62) |  | 1.79 (1.43, 2.16) |  |
| **Age, y** |  | <0.0001 |  | 0.003 |
| 40-59 | 3.05 (2.86, 3.24) |  | 2.21 (2.07, 2.35) |  |
| >60 | 2.38 (2.17, 2.59) |  | 1.89 (1.67, 2.11) |  |
| **Poverty Income Ratio, %** |  | <0.0001 |  | <0.0001 |
| <130 | 2.17 (1.92, 2.42) |  | 1.59 (1.41, 1.77) |  |
| 130-349 | 2.51 (2.28, 2.75) |  | 1.99 (1.76, 2.21) |  |
| >350 | 3.20 (2.98, 3.41) |  | 2.43 (2.24, 2.62) |  |
| Unknown | 2.98 (2.47, 3.50) |  | 1.97 (1.69, 2.25) |  |
| **Education, y** |  | 0.0006 |  | 0.006 |
| <12 | 2.42 (2.19, 2.65) |  | 1.80 (1.61, 1.99) |  |
| 12 | 2.65 (2.39, 2.91) |  | 2.06 (1.82, 2.30) |  |
| Some College, | 2.91 (2.67, 3.15) |  | 2.19 (1.97, 2.41) |  |
| >college | 3.18 (2.92, 3.44) |  | 2.29 (2.06, 2.51) |  |
| **Smoking Status** |  | 0.04 |  | 0.2 |
| Never | 3.03 (2.79, 3.27) |  | 2.16 (2.00, 2.33) |  |
| Former | 2.70 (2.53, 2.87) |  | 1.98 (1.78, 2.18) |  |
| Current | 2.69 (2.38, 2.99) |  | 2.00 (1.74, 2.26) |  |
| **Drinking status** |  | 0.002 |  | 0.1 |
| Never | 2.42 (1.97, 2.86) |  | 1.89 (1.62, 2.15) |  |
| Former | 2.51 (2.25, 2.76) |  | 2.07 (1.85, 2.29) |  |
| Current | 2.87 (2.70, 3.04) |  | 2.17 (2.01, 2.34) |  |
| Unknown | 3.27 (2.81, 3.72) |  | 1.96 (1.62, 2.30) |  |
| **BMI, kg/m^2^** |  | 0.1 |  | 0.0003 |
| <25.0 | 2.78 (2.51, 3.04) |  | 1.82 (1.62, 2.15) |  |
| 25.0-29.9 | 2.80 (2.58, 3.02) |  | 2.05 (1.85, 2.25) |  |
| >30 | 2.94 (2.70, 3.17) |  | 2.36 (2.17. 2.55) |  |
| Unknown | 2.22 (1.58, 2.86) |  | 2.12 (1.68, 2.57) |  |
| **Any leisure physical activity** |  | 0.1 |  | 0.1 |
| Yes | 2.74 (2.59, 2.88) |  | 2.15 (1.99, 2.31) |  |
| None | 2.97 (2.70, 3.25) |  | 2.00 (1.82, 2.18) |  |
| **Self-reported chronic disease** |  | 0.03 |  | 0.3 |
| Yes | 2.69 (2.50, 2.88) |  | 2.13 (1.96, 2.30) |  |
| No | 2.94 (2.76, 3.12) |  | 2.04 (1.88, 2.19) |  |

^1^Estimates are adjusted means and 95% CIs from covariate-adjusted regression models. Each estimate above was adjusted for all other variables listed in the table.
