# Supplementary material for: A prospective study of frequency of eating restaurant prepared meals and subsequent 9-year risk of all-cause and cardiometabolic mortality in US adults
Source: PLoS One. 2018 Jan 23;13(1):e0191584. doi: 10.1371/journal.pone.0191584 (PMC5779659; doi:10.1371/journal.pone.0191584)
Supplement: S2 Table — (DOCX) [file pone.0191584.s002.docx]

**S2 Table. Sensitivity analysis: ^1^covariate-adjusted hazard ratio of mortality from all-causes and cardiometabolic diseases in relation to weekly frequency of eating restaurant prepared meals after 9 years of follow-up: US men, aged >40 y at baseline**

|  | **Number of times/week eat restaurant prepared meals** | | | **P^2^** |
| --- | --- | --- | --- | --- |
|  | <1 | 1-2 | >3 (reference) |  |
| **All-cause mortality** | | | |  |
| Exclude accidental/unknown  cause of death  N=4478; events=1177 | 0.88 (0.72, 1.07) | 0.87 (0.69, 1.10) | 1.0 | 0.2 |
| Exclude first 2 y of followup  N=4248; events=985 | 0.80 (0.63, 1.01) | 0.87 (0.64, 1.16) | 1.0 | 0.1 |
| No self-reported chronic disease at base line  N=1964; events=286 | 0.70 (0.47, 1.06) | 0.90 (0.62, 1.30 | 1.0 | 0.2 |
| Reported any chronic disease at baseline  N=2514; events=928 | 0.92 (0.73, 1.15) | 0.86 (0.67, 1.1) | 1.0 | 0.3 |
| **Cardiometabolic^3^ mortality** | | | |  |
| Exclude first 2 y of followup  N=4248; events 293 | 1.15 (0.82, 1.61) | 0.84 (0.55, 1.27) | 1.0 | 0.9 |
| No self-reported chronic disease at base line  N=1964; events=64 | 1.24 (0.57, 2.73) | 1.30 (0.47, 3.55) | 1.0 | 0.5 |
| Reported any chronic disease at baseline  N=2514; events=306 | 1.35 (0.94, 1.96) | 0.84 (0.55, 1.27) | 1.0 | 0.6 |

^1^Estimates are hazard ratios and 95% CIs from Cox proportional hazards regression models. Independent variables included: number of times/week eat away from home meals (<1, 1-2, >3), race/ethnicity (non-Hispanic White, non-Hispanic Black, Mexican-American, Other), poverty income ratio, % (<130, 130-349, >350, unknown), education, y (<12, 12, some college, >college), body mass index, kg/^m2^ (<25, 25-29.9, >30, unknown), smoking status (current smoker, former smoker, never smoked), alcohol drinking status (current drinker, former drinker, never drank, unknown), self-reported doctor diagnosed chronic disease (yes, no), any leisure-time physical activity (yes, no).

^2^P value associated with weekly frequency of eating restaurant prepared meals as a trend.

^3^Cardiometabolic causes include cardiovascular and diabetes.
