# Supplementary material for: A prospective study of frequency of eating restaurant prepared meals and subsequent 9-year risk of all-cause and cardiometabolic mortality in US adults
Source: PLoS One. 2018 Jan 23;13(1):e0191584. doi: 10.1371/journal.pone.0191584 (PMC5779659; doi:10.1371/journal.pone.0191584)
Supplement: S3 Table — (DOCX) [file pone.0191584.s003.docx]

**S3 Table. Sensitivity analysis: ^1^Covariate-adjusted hazard ratio of mortality from all-causes and cardiometabolic diseases in relation to weekly frequency of eating restaurant prepared meals after 9 years of follow-up, US women, aged >40 y at baseline**

|  | **Number of times/week eat restaurant prepared meals** | | | P^2^ |
| --- | --- | --- | --- | --- |
|  | <1 | 1-2 | >3 (reference) |  |
| **All-cause mortality** | | | |  |
| Adjusted for hormone use^3^  N=4591; events 968 | 0.87 (0.66, 1.13) | 0.94 (0.70, 1.25) | 1.0 | 0.4 |
| Exclude accidental/unknown cause of death  N=4591; events=926 | 0.85 (0.65, 1.10) | 0.92 (0.70, 1.21) | 1.0 | 0.3 |
| Exclude first 2 y of followup  N=4443; events=820 | 0.88 (0.65, 1.18) | 0.97 (0.72, 1.30) | 1.0 | 0.5 |
| No self-reported chronic disease at base line  N=1904; events=208 | 0.90 (0.61, 1.35) | 1.02 (0.64, 1.64) | 1.0 | 0.8 |
| Reported any chronic disease at baseline  N=2687; events 760 | 0.87 (0.65, 1.16) | 0.91 (0.68, 1.23) | 1.0 | 0.4 |
| **Cardiometabolic^4^ mortality** | | | |  |
| Adjusted for hormone use^3^  N=4591; events 291 | 0.74 (0.44, 1.22) | 0.84 (0.51, 1.38) | 1.0 | 0.3 |
| Exclude first 2 y of followup  N=4443; events 241 | 0.80 (0.47, 1.36) | 1.05 (0.65, 1.70) | 1.0 | 0.6 |
| No self-reported chronic disease at base line  N=1904; events=54 | 0.64 (0.27, 1.51) | 0.59 (0.22, 1.62) | 1.0 | 0.2 |
| Reported any chronic disease at baseline  N=2687; events 237 | 0.74 (0.43, 1.27) | 0.86 (0.45, 1.43) | 1.0 | 0.3 |

^1^Estimates are hazard ratios and 95% CIs from Cox proportional hazards regression models. Independent variables included: number of times/week eat away from home meals (<1, 1-2, >3), race/ethnicity (non-Hispanic White, non-Hispanic Black, Mexican-American, Other), poverty income ratio, % (<130, 130-349, >350, unknown), education, y (<12, 12, some college, >college), body mass index, kg/^m2^ (<25, 25-29.9, >30, unknown), smoking status (current smoker, former smoker, never smoked), alcohol drinking status (current drinker, former drinker, never drank, unknown), self-reported doctor diagnosed chronic disease (yes, no), any leisure-time physical activity (yes, no).

^2^P value associated with weekly frequency of eating restaurant prepared meals as a trend.

^3^Hormone use: self-reported history of non-oral contraceptive hormone use (yes, no).

^4^Cardiometabolic causes include cardiovascular and diabetes.
